# Optimizing Laboratory Autoverification in Complete Blood Count Testing Using Machine Learning: A Performance Evaluation Study

**DOI:** 10.1002/jcla.70335

**Published:** 2026-08-11

**Authors:** Sinsorn Srirujee, Peempol Chokchaipermpoonphol

**Affiliations:** ^1^ Division of Information Technology, Faculty of Medicine Prince of Songkla University Songkhla Thailand; ^2^ Department of Pathology, Faculty of Medicine Prince of Songkla University Songkhla Thailand

**Keywords:** complete blood count, decision tree, extreme gradient boost, laboratory autoverification, machine learning, random forest

## Abstract

**Background:**

Autoverification improves laboratory efficiency by reducing manual reviews; however, rule‐based systems may lack flexibility, particularly in complete blood count (CBC) testing. Machine learning (ML) can potentially enhance performance by recognizing complex patterns in laboratory data. Therefore, we aimed to evaluate ML‐based autoverification systems in classifying CBC results compared to traditional rule‐based methods.

**Methods:**

This cross‐sectional study was conducted at the Hematology Unit of Songklanagarind Hospital, a tertiary care center in southern Thailand. In total, 63,201 CBC results from the Sysmex XN‐10 analyzer (January–December 2024) were analyzed. The samples were categorized as verifiable or non‐verifiable according to predefined criteria. The data were split 80:20 into training and testing sets. The Random Forest, Decision Tree, and Extreme Gradient Boost models were trained and evaluated against the rule‐based system of the hospital using sensitivity, specificity, positive predictive value, and negative predictive value. The feature importance was analyzed for the best‐performing model.

**Results:**

Of all the results, 43,661 (69.1%) were verifiable and 19,540 (30.9%) were non‐verifiable, mainly because of white blood cell abnormalities, platelet clumps, or differential discrepancies. In the 12,641‐sample test set, all the ML models outperformed the rule‐based system. XGBoost achieved the best balance, with a sensitivity of 95%–98% and a specificity of 68%–81% at recall thresholds of 95% and 97.5%, respectively.

**Conclusion:**

ML demonstrated improved performance compared with rule‐based autoverification. Although ML may reduce unnecessary manual reviews, validation is required in different settings before clinical adoption.

## Graphical abstract

This cross‐sectional study evaluated the feasibility of applying machine learning for complete blood count autoverification. Among 63,201 CBC results, the XGBoost model achieved higher sensitivity and predictive values compared with the traditional rule‐based system.

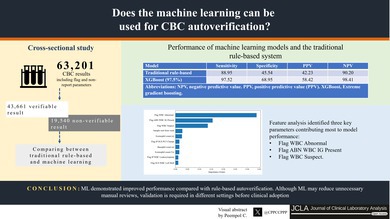

## 1 Introduction

In modern clinical laboratories, the increasing volume of tests and complexity of data require efficient and reliable systems to ensure accurate results. Autoverification, a process that automatically validates laboratory test results based on predetermined criteria, has emerged as a critical tool for enhancing workflow efficiency and reducing human error [1]. Traditional rule‐based auto‐verification systems have been successfully implemented and yielded reliable results [2, 3, 4]. However, they may still lead to false rejections, requiring manual review and potentially delaying the reporting of results. Moreover, they may lack the flexibility to adapt to nuanced patterns in patient data, particularly with complex parameters, such as those of complete blood count (CBC) analyses.

Machine learning (ML) has been successful in several laboratory testing aspects. In clinical biochemistry, ML‐based ensemble models achieve an 89.6% passing rate with very low false negatives and reduce invalid reports by nearly 80% compared with traditional rule‐based systems [5]. More recently, ML has been applied to detect sample misidentification errors in over 600,000 cases, where advanced models such as deep neural networks and extreme gradient boosting have significantly outperformed conventional delta‐check methods and maintained strong accuracy across multiple laboratories [6]. These successes highlight the potential of ML in improving the accuracy, adaptability, and operational efficiency of laboratory medicine.

The CBC is a high‐volume test that includes numerous numerical and categorical parameters, such as measured values, system‐generated flags, and instrument‐derived quality indicators that are often not reported to clinicians. These rich and multifaceted data elements provide a strong foundation for ML applications. Integrating ML into the autoverification of CBC results could enable a more precise interpretation of complex patterns, reduce unnecessary manual reviews, and improve both workflow efficiency and patient care outcomes.

This study aimed to evaluate the ability of ML‐based autoverification systems to accurately classify CBC results as either valid or requiring further review, compared to traditional rule‐based methods. Additionally, it seeks to identify predictive features and patterns within CBC data that are most strongly associated with aberrant or review‐worthy results to refine and optimize model performance.

## 2 Materials and Methods

This cross‐sectional study was conducted at the Hematology Unit, Clinical Pathology Division, Department of Pathology, Songklanagarind Hospital, a tertiary care hospital in southern Thailand. The data collection period included CBC results recorded between January and December 2024. The inclusion criteria were all CBC results generated using the Sysmex XN‐10 hematology analyzer during the study period. The exclusion criteria were records with incomplete data or missing variables required for model development and performance evaluation.

The dataset included both reported and non‐reported CBC parameters directly obtained from the analyzer. The hematological parameters included RBC indices, including hemoglobin (Hb), hematocrit (Hct), mean corpuscular volume (MCV), mean corpuscular hemoglobin (MCH), mean corpuscular hemoglobin concentration (MCHC), and red cell distribution width (RDW); WBC and total nucleated cell (TNC) counts; platelet count; and five‐part differential counts, including neutrophils, lymphocytes, monocytes, eosinophils, and basophils, reported as both absolute values and percentages. Additional hematological parameters included nucleated RBC (NRBC) count and immature granulocyte percentage (%IG).

The dataset also included instrument‐generated flags related to WBC, RBC, and platelet abnormalities. WBC‐related flags included Blasts?, Abn Lympho?, Left Shift?, Atypical Lympho?, NRBC Present, and WBC Abn Scattergram. RBC‐related flags included RBC Agglutination?, HGB Defect?, RBC Fragments?, and Dimorphic Population. Platelet‐related flags included PLT Clumps?, Abnormal PDW, and PLT Abn Scattergram. In addition, non‐reportable analyzer‐derived parameters, including WDF‐X/Y/Z, WNR‐X/Y/Z, IG‐X/Y/Z, DIFF‐WX/WY/WZ, and cell population‐specific width parameters such as Ne‐WX, Mo‐WY, and BASO‐WZ, were extracted for model development.

Demographic data, including patient age, sex, and care setting (outpatient and inpatient) were also collected. To assess the autoverification performance, the corresponding CBC reports in the hospital information system were reviewed. Each case was classified as verifiable or non‐verifiable. Results were defined as non‐verifiable if they met any of the following criteria: (1) presence of immature granulocytes (including blasts, promyelocytes, myelocytes, or metamyelocytes); (2) presence of atypical lymphocytes; (3) platelet clumps or clotting observed; or (4) difference in the WBC differential result from the manual differential beyond the acceptable limits of analytical bias as defined by the state‐of‐the‐art criteria in hematology based on the Clinical and Laboratory Standards Institute H26‐A2 guidelines [7]. The acceptable bias goals were 5.0% for neutrophils, 12.0% for lymphocytes, 26.0% for monocytes, 29.0% for eosinophils, and 42.0% for basophils.

Statistical analyses were performed using Python (Python Software Foundation, Wilmington, DE, USA) on Google Colab (Google, Mountain View, CA, USA). Data were first summarized using descriptive statistics, with means and standard deviations reported for continuous variables and frequencies with percentages for categorical variables. Analyzer‐generated flags were entered as individual variables in the model without enforcing predefined group‐level assumptions, enabling a data‐driven learning process consistent with machine learning principles. The dataset was then split into training and testing subsets at an 80:20 ratio. ML models, including Random Forest (RF), Decision Tree (DT), and Extreme Gradient Boosting (XGBoost) were trained on the training set. The model performance was evaluated on the test set, with an emphasis on maintaining acceptable false negative (recall) thresholds at 95% and 97.5%. No resampling or class‐weighting techniques were applied; instead, model evaluation focused on predefined high‐recall thresholds to reflect clinically relevant operating conditions for autoverification. Confidence intervals (CIs) for sensitivity (recall), specificity, positive predictive value (PPV), and negative predictive value (NPV) were calculated using the Wilson score method for binomial proportions based on the confusion matrix obtained from the independent test set.

The required sample size for recall estimation was determined based on the standard binomial formulation for estimating a proportion, in which the expected half‐width of the confidence interval is given by $E=z\sqrt{\frac{p\left(1-p\right)}{n}}$, where *p* denotes the target recall, *n* is the required number of positive cases, and *z* = 1.96 for a 95% confidence level. Rearranging the equation yields $n=\frac{z^{2}p\left(1-p\right)}{E^{2}}$. Using this formulation, the required numbers of positive cases were approximately 1825 for a target recall of 95% and 936 for a target recall of 97.5%, assuming a desired half‐width (*E*) of 0.01 for the 95% confidence interval.

The evaluation metrics included sensitivity, specificity, positive predictive value (PPV), and negative predictive value (NPV). These outcomes were compared with those of the traditional rule‐based autoverification system currently implemented at Songklanagarind Hospital. The hospital's criteria define acceptable results as follows: for WBC differential, results must fall within 3.0–15.0 × 10^3^/μL without warning messages (ALT, Blast, or Left shift), with a clearly separated scattergram, and lymphocytes ≤ 55%, monocytes ≤ 12%, and immature granulocytes (IG) ≤ 1.5%. For RBC morphology, results are valid when hematocrit is ≥ 36%, with no RBC IP messages and a normal RBC scattergram. For platelet counts, valid results are defined as 130–500 × 10^3^/μL without IP messages and with a normal platelet scattergram. The ML model demonstrating the highest sensitivity and specificity was further analyzed to investigate feature importance for identifying the most influential parameters contributing to autoverification decisions.

The study protocol was reviewed and approved by the Human Research Ethics Committee of the Faculty of Medicine, Prince of Songkla University, Thailand (REC 68‐077‐5‐1). To protect participants' confidentiality, all data were anonymized prior to analysis. The study involved no additional risk as analyses were performed exclusively on de‐identified samples, and the findings did not influence patient management or clinical care.

## 3 Results

Over the 1‐year study period, 63,201 CBC results were included in the analysis. Based on predefined verification criteria, 43,661 results were classified as verifiable. Of the 19,540 cases classified as non‐verifiable, the main causes of rejection were: 358 cases had blasts, 4411 cases had immature granulocytes, 1818 cases had platelet clumps or clotting, and 6049 cases had atypical lymphocytes. In addition, 18,703 cases were rejected because the WBC differential showed discrepancies beyond the acceptable percentage difference compared with the manual differential. Some patients exhibited more than one cause of rejection. Overall, abnormalities related to WBC differential interpretation represented the predominant reason for non‐verifiable CBC results in this study. The detailed distribution of the demographic characteristics of the study population is presented in Table 1, and the distribution of analyzer‐generated flags and non‐reported parameters is provided in Table S1. These results indicate that samples from hospital wards were more frequently classified as non‐verifiable than verifiable. In contrast, verifiable cases were associated with higher hemoglobin, hematocrit, RBC, and platelet counts, and slightly lower MCHC. Other red blood cell indices and differential counts showed no significant differences between the groups.

**TABLE 1:** Baseline demographic characteristics of complete blood count results categorized as verifiable and non‐verifiable.

Variable	Non‐verifiable result	Verifiable result
*N*	19,540	43,661
Age	50.38 ± 26.56	57.48 ± 19.06
Sex (male)	9175 (47.0%)	19,223 (44.0%)
Sample sent from ward	10,123 (51.8%)	7674 (17.6%)
Hb, g/dL	10.56 ± 2.40	12.03 ± 2.02
Hct, %	32.18 ± 7.08	36.97 ± 5.87
RBC, ×10^6^ cells/μL	3.86 ± 0.93	4.35 ± 0.76
MCV, fL	84.26 ± 9.16	85.54 ± 8.23
MCH, pg	27.68 ± 3.44	27.84 ± 3.13
MCHC, g/dL	32.82 ± 1.55	32.51 ± 1.25
RDW, %	16.04 ± 3.42	14.51 ± 2.40
WBC, ×10^3^ cells/μL	9.77 ± 16.14	7.42 ± 2.95
%Neutrophils	65.83 ± 20.29	61.15 ± 12.75
%Eosinophils	3.60 ± 4.04	3.64 ± 3.78
%Basophils	1.16 ± 1.16	0.56 ± 0.32
%Lymphocytes	22.71 ± 17.64	28.55 ± 11.60
%Monocytes	6.50 ± 4.32	6.10 ± 2.01
PLT	239.10 ± 147.45	261.08 ± 94.84

*Note:* Continuous variables are represented as means (SD), while dichotomous variables are represented as numbers (%).

Abbreviations: Hb, hemoglobin; Hct, hematocrit; MCH, mean corpuscular hemoglobin; MCHC, mean corpuscular hemoglobin concentration; MCV, mean corpuscular volume; RBC, red blood cell count; WBC, white blood cell count.

For the 12,641 samples in the testing set, the performance of each ML model was compared with that of the traditional rule‐based system, as summarized in Table 2. The traditional rule‐based system showed limited overall performance, with lower specificity and predictive values, indicating a higher burden of unnecessary manual review. In contrast, all machine learning models demonstrated improved performance across the evaluated metrics. At the 95% recall threshold, XGBoost achieved a sensitivity of 95.04%, specificity of 81.46%, PPV of 69.64%, and NPV of 97.35%. At the 97.5% recall threshold, XGBoost maintained high sensitivity at 97.52%, with a specificity of 68.95%, PPV of 58.42%, and NPV of 98.41%. Among the tested models, XGBoost yielded the best overall performance at both 95% and 97.5% recall thresholds, superiorly balancing between sensitivity and specificity while maintaining high predictive values.

**TABLE 2:** Performance of machine learning models and the traditional rule‐based system in classifying verifiable and non‐verifiable complete blood count results.

Model	Sensitivity	Specificity	PPV	NPV
Traditional rule‐based	88.95 (87.92–89.89)	45.54 (44.50–46.59)	42.23 (41.16–43.30)	90.20 (89.29–91.04)
Random forest (95%)	95.01 (94.28–95.65)	79.80 (78.95–80.63)	67.79 (66.54–69.02)	97.28 (96.88–97.63)
Random forest (97.5%)	97.52 (96.98–97.96)	66.72 (65.73–67.70)	56.74 (55.55–57.92)	98.36 (98.01–98.66)
Decision tree (95%)	95.42 (94.72–96.03)	73.39 (72.45–74.30)	61.61 (60.37–62.82)	97.28 (96.86–97.65)
Decision tree (97.5%)	98.16 (97.69–98.53)	40.03 (39.01–41.06)	42.28 (41.27–43.30)	97.98 (97.47–98.39)
XGBoost (95%)	95.04 (94.31–95.67)	81.46 (80.63–82.26)	69.64 (68.39–70.86)	97.35 (96.95–97.69)
XGBoost (97.5%)	97.52 (96.98–97.96)	68.95 (67.97–69.91)	58.42 (57.22–59.61)	98.41 (98.07–98.70)

*Note:* All results are shown as percentages with 95% CI.

Abbreviations: NPV, negative predictive value; PPV, positive predictive value (PPV); XGBoost, extreme gradient boosting.

**FIGURE 1:**
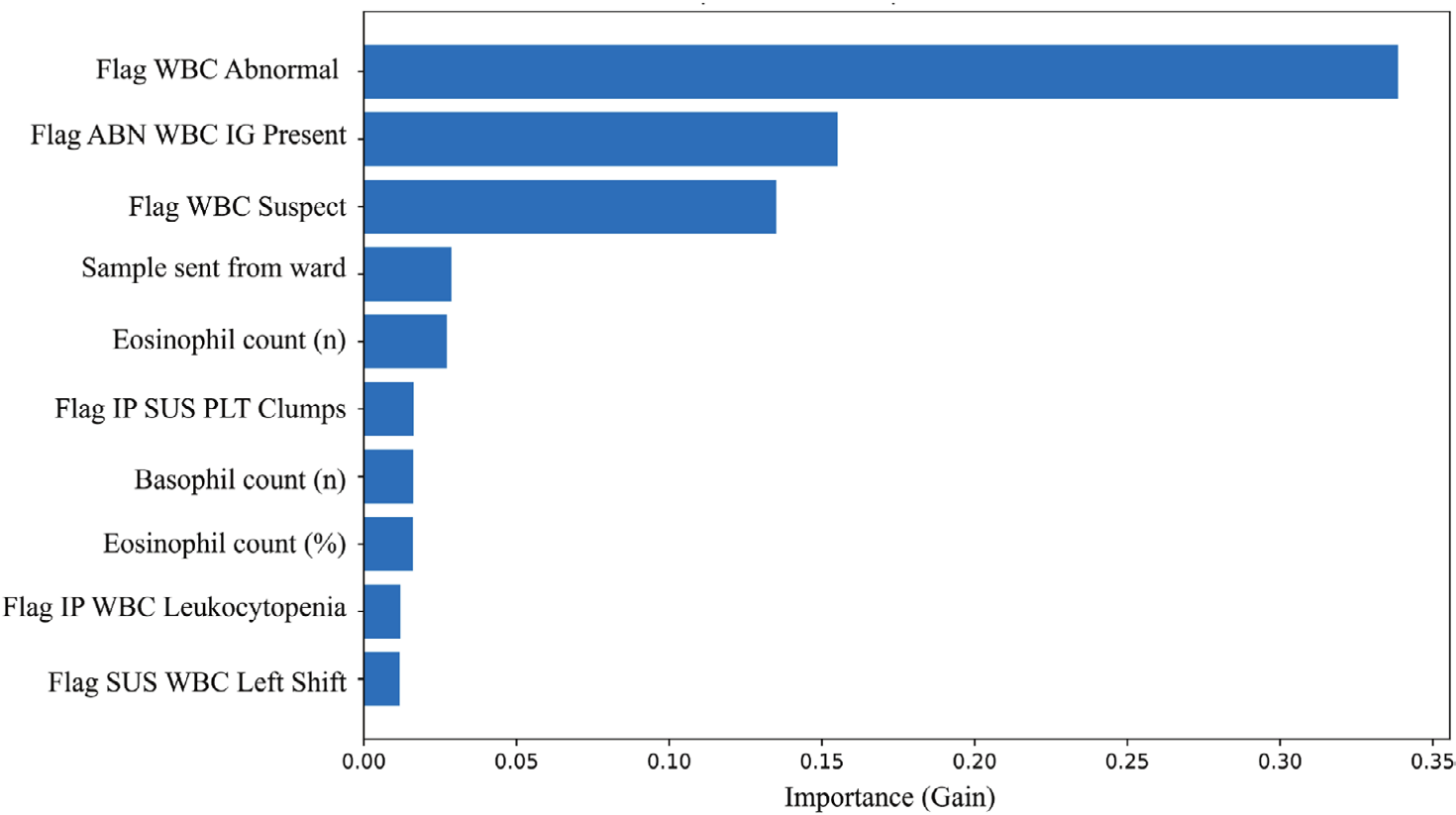
Feature importance according to the XGBoost model for the autoverification of complete blood count results.

The feature importance was subsequently evaluated using the XGBoost model, which demonstrated the highest overall performance. In XGBoost, feature importance is derived from the contribution of each variable to tree‐based splits, and it is quantified by the improvement in model performance (gain) when the feature is used. The top ten contributing features are shown in Figure 1. The three most influential features were *Flag WBC Abnormal*, *Flag ABN WBC IG Present*, and *Flag WBC Suspect*.

## 4 Discussion

This study demonstrated the feasibility and potential advantages of integrating ML into the autoverification of CBC results. Over 1 year, more than 63,000 CBC reports were analyzed, with approximately one‐third classified as non‐verifiable according to predefined criteria. The main causes of rejection were the presence of abnormal white cell populations, platelet clumping, and discrepancies in WBC differentials compared with the manual review. When benchmarked against the traditional rule‐based system currently used at Songklanagarind Hospital, all ML models tested in this study exhibited superior performance with higher sensitivity, specificity, and predictive values. XGBoost provided the most balanced tradeoff between sensitivity and specificity at the chosen recall thresholds, with analyzer‐generated flags contributing the most strongly to the classification. Although the specificity gains observed in our setting may not translate directly to others, the results suggest that existing instrument‐derived signals remain highly influential and may be better leveraged through ML approaches.

Our results are consistent with those of previous research, demonstrating that autoverification systems can reduce false alarms and unnecessary manual review. For instance, in a study by Nuanin et al., an auto‐verification system achieved 100% sensitivity and 77.6% specificity, corresponding to a 22% false‐positive rate [8]. In comparison, our ML models showed substantially improved specificity while maintaining high sensitivity, indicating fewer false alarms. Compared with the traditional rule‐based system, the main advantage of the ML‐based approach was its ability to integrate multiple CBC parameters, analyzer‐generated flags, and non‐reportable instrument‐derived signals simultaneously, thereby reducing unnecessary manual review while preserving a high sensitivity for non‐verifiable results. The decreased false‐alarm burden of ML models strengthens their practical appeal, particularly in high‐throughput hematology settings, by reducing the manual review workload.

Although ML applications in laboratory medicine remain relatively limited, early clinical chemistry studies have supported their potential. Wang et al. demonstrated that ML‐based ensemble models for biochemistry testing achieved higher pass rates and markedly reduced the number of invalid reports compared with rule‐based methods [5]. Similarly, Zhu et al. proposed a combined strategy integrating knowledge‐based rules with ML models to achieve improved performance and interpretability [9]. Our findings align with these reports, suggesting that ML‐driven autoverification reduces unnecessary manual reviews and improves overall efficiency in hematology while retaining interpretability through evaluating feature importance.

Recent studies have directly applied ML to CBC data to detect abnormal patterns. Fang et al. developed a convolutional neural network model using Sysmex scattergram images to identify platelet clumps, reporting a sensitivity and specificity above 90%, outperforming the analyzer's internal flagging algorithm [10]. Similarly, Amjad et al. used ML models trained on routine CBC parameters to classify hematological disorders such as anemia or leukocyte abnormalities, demonstrating that standard hematological data contain predictive information beyond fixed thresholds [11]. These examples support the applicability of ML in hematology and align with our observation that analyzer‐generated flags and abnormal cell patterns were the most influential predictors of autoverification.

XGBoost exhibited the highest performance, with superior sensitivity and specificity at both recall thresholds. It is a gradient‐boosting algorithm that builds decision trees sequentially, with each tree correcting errors from the previous ones. At every split, the model measures how much the accuracy has improved, known as gain, and aggregates these values across all trees to estimate the feature importance [12]. In this study, *Flag WBC Abnormal*, *Flag ABN WBC IG Present*, and *Flag WBC Suspect* contributed to the highest gains, highlighting their strong influence on distinguishing verifiable CBC results. However, a large proportion of cases classified as non‐verifiable were associated with abnormalities in the WBC differential, such as the presence of blasts. Therefore, this aggregation of abnormal WBC findings may influence the relative contribution of these features to model performance.

For sustainable implementation in routine laboratory practice, machine learning–based autoverification systems require ongoing monitoring to detect performance degradation and guide retraining decisions. Recent work emphasizes that deployed predictive models are subject to dataset and concept drift, which can lead to temporal deterioration in model accuracy if unaddressed [13]. In the clinical laboratory context, best practices for validating, implementing, and monitoring ML applications include routine performance surveillance and updating strategies to mitigate model decay [14]. Accordingly, the present study frames ML‐based autoverification not primarily as an exercise in maximizing analytical performance, but as a workflow‐oriented decision‐support tool evaluated under predefined high‐recall operating points aligned with laboratory safety requirements. By examining rejection rates, false‐negative behavior, and implications for manual review workload, this approach emphasizes operational efficiency and scalability alongside analytical validity. Reviews of machine learning operations in healthcare underscore the importance of model monitoring, retraining triggers, and maintenance within clinical workflows [15]. A systematic approach that combines distributional monitoring, periodic performance assessment, and retraining triggered by meaningful deviations aligns with emerging standards for reliable and scalable ML deployment in medical environments.

This study had some limitations. First, the study was conducted at a single institution, which may limit the generalizability of the findings to other laboratory settings. Second, differences in rule‐based criteria, causes of rejection, and laboratory practices across institutions may have influenced the performance and applicability of the proposed model. Moreover, the non‐verifiable criteria used in this study did not encompass all potential analytical or pre‐analytical interferences. Hemolysis, icterus, and lipemia (HIL) were not included because CBC analysis is performed on whole blood and the hematology analyzer does not routinely provide HIL indices, while detailed schistocyte data were unavailable in this retrospective dataset. In addition, the study did not incorporate delta check rules, which are an essential component of autoverification, because of the limited ability of the current machine learning framework to handle missing and incomplete data. Nonetheless, each laboratory is required to establish and validate its own autoverification system according to its workflow and patient population, which is consistent with the individualized approach recommended by international guidelines. Third, the study did not evaluate the long‐term stability or the need for continuous retraining of ML models, which are essential for sustainable implementation. Fourth, cross‐validation or class‐balancing techniques were not employed; instead, evaluation focused on predefined high‐recall thresholds using an independent test set to reflect clinically relevant autoverification conditions. Future studies may explore imbalance‐aware strategies to further enhance robustness. Finally, clinical outcomes were not assessed; therefore, the impact of the model on patient care and diagnostic accuracy remains to be determined. Future studies should extend this work by validating the model across multiple institutions and analyzer platforms to ensure generalizability, while also exploring cause‐specific modeling to determine whether tailored approaches improve the performance for different types of rejection. Prospective investigations are needed to assess the stability of the model performance over time and to establish effective strategies for periodic retraining with new data. Further research should examine the clinical impact of ML‐based autoverification on turnaround time, workload reduction, and patient outcomes.

In conclusion, this study showed that ML models, especially XGBoost, performed better than the traditional rule‐based system in classifying CBC results at our institution. Although performance improvements were observed, the benefit was moderate. Analyzer‐generated flags such as WBC Abnormal, IG Present, and WBC Suspect were the most influential features. These findings indicate that ML can complement existing rule‐based autoverification systems in hematology by modestly improving specificity. However, the clinical impact of the reduced manual review workload or turnaround time was not measured in this study and warrants further investigation. Further multicenter studies are needed to confirm generalizability and clinical utility.

## Conflicts of Interest

The authors declare no conflicts of interest.

## Supporting information


**Table S1:** Distribution of Sysmex XN‐10 analyzer–generated flags and non‐report parameters.

## Data Availability

The code used for data preprocessing, model training, and evaluation is available at: https://github.com/cpeempol/CBC‐autoverification‐ML. Patient‐level data are not publicly available due to institutional data protection policies.
