# Supplementary material for: Optimizing Laboratory Autoverification in Complete Blood Count Testing Using Machine Learning: A Performance Evaluation Study
Source: J Clin Lab Anal. 2026 Aug 11;40(18):e70335. doi: 10.1002/jcla.70335 (PMC13458867; doi:10.1002/jcla.70335)
Supplement: Supplementary file 1 [file JCLA-40-e70335-s001.docx]

Supplementary S1

Table S1: Distribution of Sysmex XN-10 analyzer–generated flags and non-report parameters

| Variable | Non-verifiable result | Verifiable result |
| --- | --- | --- |
| Flag | | |
| Q Flag LeftShift | 47.29±82.31 | 4.72±13.77 |
| Q Flag Atypical Lympho | 66.88±83.87 | 20.60±27.38 |
| Q Flag RBC Agglutination | 64.10±11.26 | 64.10±8.25 |
| Q Flag TurbidityHGBInterf | 85.17±8.84 | 83.79±5.33 |
| Q Flag Iron Deficiency | 81.90±7.97 | 81.46±7.25 |
| Q FlagHGB Defect | 79.03±10.35 | 80.58±7.68 |
| Q Flag Fragments | 19.07±39.91 | 7.24±22.14 |
| Q Flag PLT Clumps | 53.55±85.86 | 26.86±50.70 |
| WBC Abnormal | 12033 (61.6%) | 3921 (9.0%) |
| WBC Suspect | 10660 (54.6%) | 2147 (4.9%) |
| RBC Abnormal | 13219 (67.7%) | 16430 (37.6%) |
| RBC Suspect | 2235 (11.4%) | 3034 (6.9%) |
| PLT Abnormal | 7342 (37.6%) | 5339 (12.2%) |
| PLT Suspect | 3266 (16.7%) | 2635 (6.0%) |
| IP ABN WBC Abn Scattergram | 1563 (8.0%) | 22 (0.1%) |
| IP ABN WBC Lymphocytosis | 2021 (10.3%) | 384 (0.9%) |
| IP ABN WBC Monocytosis | 2066 (10.6%) | 589 (1.3%) |
| IP ABN WBC Leukocytopenia | 2236 (11.4%) | 274 (0.6%) |
| IP ABN WBC Leukocytosis | 2610 (13.4%) | 1039 (2.4%) |
| IP ABN RBC RBC Abn Distribution | 455 (2.3%) | 232 (0.5%) |
| IP ABN RBC Anisocytosis | 7453 (38.1%) | 7869 (18.0%) |
| IP ABN RBC Microcytosis | 5479 (28.0%) | 9207 (21.1%) |
| IP ABN RBC Macrocytosis | 666 (3.4%) | 833 (1.9%) |
| IP ABN RBC Hypochromia | 48 (0.2%) | 51 (0.1%) |
| IP ABN RBC Anemia | 8389 (42.9%) | 6852 (15.7%) |
| IP ABN RBC Erythrocytosis | 144 (0.7%) | 342 (0.8%) |
| IP ABN RBC RET Abn Scattergram | 147 (0.8%) | 42 (0.1%) |
| IP ABN RBC Reticulocytosis | 282 (1.4%) | 56 (0.1%) |
| IP ABN PLT PLT Abn Distribution | 2896 (14.8%) | 2017 (4.6%) |
| IP ABN PLT Thrombocytopenia | 4791 (24.5%) | 2120 (4.9%) |
| IP ABN PLT Thrombocytosis | 1452 (7.4%) | 1679 (3.8%) |
| IP SUS WBC Blasts Abn Lympho | 5101 (26.1%) | 937 (2.1%) |
| IP SUS WBC Left Shift | 3072 (15.7%) | 80 (0.2%) |
| IP SUS RBC RBC Agglutination | 78 (0.4%) | 16 (0.0%) |
| IP SUS RBC Iron Deficiency | 693 (3.5%) | 1628 (3.7%) |
| IP SUS RBC HGB Defect | 695 (3.6%) | 1076 (2.5%) |
| IP SUS PLT PLT Clumps | 3266 (16.7%) | 2635 (6.0%) |
| Non-report parameters | | |
| HFLC | 0.05±0.16 | 0.01±0.02 |
| HFLCper | 0.61±1.68 | 0.18±0.22 |
| BA-D | 0.04±0.18 | 0.04±0.02 |
| NE-SSC | 152.74±7.32 | 153.50±4.22 |
| NE-SFL | 50.89±9.33 | 48.04±4.48 |
| NE-FSC | 84.68±6.97 | 86.72±3.67 |
| LY-X | 77.42±4.63 | 76.58±2.24 |
| LY-Y | 67.71±9.59 | 67.46±5.96 |
| LY-Z | 56.04±2.16 | 55.74±1.42 |
| MO-X | 119.33±5.31 | 118.24±2.28 |
| MO-Y | 112.85±16.37 | 112.23±9.93 |
| MO-Z | 64.18±4.15 | 65.08±1.98 |
| NE-WX | 320.35±51.52 | 303.01±18.28 |
| NE-WY | 682.85±197.58 | 602.09±41.19 |
| NE-WZ | 666.14±91.10 | 649.35±48.55 |
| LY-WX | 580.05±121.06 | 552.59±60.73 |
| LY-WY | 939.16±253.65 | 868.82±98.39 |
| LY-WZ | 581.20±122.46 | 534.12±66.92 |
| MO-WX | 267.19±58.21 | 259.65±25.56 |
| MO-WY | 658.77±188.86 | 665.89±89.25 |
| MO-WZ | 590.91±117.39 | 579.07±68.70 |
